# Global Alterations of Whole Brain Structural Connectome in Parkinson’s Disease: A Meta-analysis

**DOI:** 10.1007/s11065-022-09559-y

**Published:** 2022-09-20

**Authors:** Chao Zuo, Xueling Suo, Huan Lan, Nanfang Pan, Song Wang, Graham J. Kemp, Qiyong Gong

**Affiliations:** 1 Huaxi MR Research Center (HMRRC), Department of Radiology, West China Hospital of Sichuan University, Chengdu, Sichuan China; 2 Liverpool Magnetic Resonance Imaging Centre (LiMRIC) and Institute of Life Course and Medical Sciences, University of Liverpool, Liverpool, UK; 3 Research Unit of Psychoradiology, Chinese Academy of Medical Sciences, Chengdu, Sichuan China; 4 Functional and Molecular Imaging Key Laboratory of Sichuan Province, West China Hospital of Sichuan University, Chengdu, Sichuan China; 5 Department of Radiology, West China Xiamen Hospital of Sichuan University, Xiamen, Fujian, China

**Keywords:** Parkinson’s disease, Structural connectome, Diffusion MRI, Psychoradiology, Graph theory, Meta-analysis

## Abstract

Recent graph-theoretical studies of Parkinson's disease (PD) have examined alterations in the global properties of the brain structural connectome; however, reported alterations are not consistent. The present study aimed to identify the most robust global metric alterations in PD via a meta-analysis. A comprehensive literature search was conducted for all available diffusion MRI structural connectome studies that compared global graph metrics between PD patients and healthy controls (HC). Hedges’ g effect sizes were calculated for each study and then pooled using a random-effects model in Comprehensive Meta-Analysis software, and the effects of potential moderator variables were tested. A total of 22 studies met the inclusion criteria for review. Of these, 16 studies reporting 10 global graph metrics (916 PD patients; 560 HC) were included in the meta-analysis. In the structural connectome of PD patients compared with HC, we found a significant decrease in clustering coefficient (g = -0.357, P = 0.005) and global efficiency (g = -0.359, P < 0.001), and a significant increase in characteristic path length (g = 0.250, P = 0.006). Dopaminergic medication, sex and age of patients were potential moderators of global brain network changes in PD. These findings provide evidence of decreased global segregation and integration of the structural connectome in PD, indicating a shift from a balanced small-world network to ‘weaker small-worldization’, which may provide useful markers of the pathophysiological mechanisms underlying PD.

**Supplementary Information:**

The online version contains supplementary material available at 10.1007/s11065-022-09559-y.

## Introduction

Parkinson’s disease (PD) is a common, complex, progressive multi-system neurodegenerative disease mainly affecting older people (Kalia & Lang, 2015; Pringsheim et al., 2014), and is the fastest growing of the leading neurological causes of disability (Dorsey et al., 2018). The symptoms of PD are generally classified into motor symptoms (including bradykinesia, resting tremor, and postural and gait impairment) and non-motor symptoms (such as disturbances in autonomic function, sleep disturbances, cognitive and psychiatric disturbances, and sensory symptoms) (Kalia & Lang, 2015; Sveinbjornsdottir, 2016). Neuroimaging biomarkers including cortical thickness (a structural marker) and dopaminergic imaging of the striatum (a PET imaging methodology, yielding a functional measure) have been used clinically for early diagnosis, prognosis and disease course management (Mitchell et al., 2021). However, the underlying neurobiology has not been fully elucidated.

Advanced magnetic resonance imaging (MRI) techniques are increasingly used to investigate the pathophysiology of neurodegenerative diseases (Cerasa et al., 2012; Kassubek & Müller, 2016; Suo et al., 2019; Weingarten et al., 2015). Previous studies in PD have focused on specific regions of interest such as substantia nigra (Deng et al., 2018; Hirata et al., 2017; Ofori et al., 2015) and basal ganglia (Fioravanti et al., 2015). However, localized brain alterations are not sufficient to explain the clinical heterogeneity (Rodriguez-Oroz et al., 2009). It is now clear that PD involves altered connections between various brain regions and can therefore be considered a network-disconnection syndrome (Cronin-Golomb, 2010; Nigro et al., 2016; Premi et al., 2016).

Brain connectivity studies are of two main kinds: of structural connectivity based on data from structural MRI or diffusion MRI (dMRI); and of functional connectivity using data from functional MRI (Zhang et al., 2022), electroencephalography, or magnetoencephalography. Neuroimaging studies have reported abnormal brain connectivity in PD patients (Hall et al., 2016; Weingarten et al., 2015) e.g. in basal ganglia circuits (Wu et al., 2012), in cortico–basal ganglia–thalamo-cortical circuits (Rodriguez-Oroz et al., 2009; Singh, 2018), and between basal ganglia and motor regions (Helmich et al., 2010). However, pathological changes in PD are not restricted to isolated brain regions, and no model with separate analyses of different regions or a single neural circuit can account for the whole clinical and behavioral spectrum.

Graph theoretical analysis (GTA) allows analysis of the topological properties of the interconnected whole-brain network (the connectome) and quantification of its abnormalities in vivo (Bullmore & Sporns, 2009; Griffa et al., 2013; Rubinov & Sporns, 2010). The brain is modeled as a large-scale network composed of nodes (brain regions) and edges (connections between nodes) (Suo et al., 2022a). The nodes represent cortical and subcortical regions, determined by a specific a priori template, the edges reflecting either their structural or functional connections. As white matter tracts are the key components of these large-scale distributed networks, to limit cross-study heterogeneity we focus our analysis on structural connectivity measured by dMRI. Tractography, a key method in ‘connectomics’, infers continuity of white matter tracts from voxel to voxel (Jeurissen et al., 2019). Brain network topology can be quantified by a variety of metrics summarised in Supplementary Table S1, including measures of global segregation, global integration and small-worldness (σ) (Sporns, 2013; Watts & Strogatz, 1998): network integration denotes the ability to transfer information rapidly between different nodes, and its metrics are characteristic path length (L_p_), global efficiency (E_glob_) and normalized characteristic path length (λ); network segregation denotes ability to perform specific processing in closely-interconnected clusters of brain regions, and its metrics are clustering coefficient (C_p_), normalized clustering coefficient (γ) and local efficiency (E_loc_); finally σ is the ratio of γ to λ, and represents the balance between network segregation and network integration. GTA has been widely applied (Sanz-Arigita et al., 2010; Suo et al., 2017, 2018, 2022) and shows promise in neuropsychiatric disorders (Griffa et al., 2013) such as traumatic brain injury (Imms et al., 2019), Alzheimer’s disease (Yu et al., 2021) and schizophrenia (Zhao et al., 2018). Several studies of PD have reported topological changes in the structural connectome compared to healthy controls (HC) (Abbasi et al., 2018; Galantucci et al., 2017; Kamagata et al., 2017). However, these are not consistent, and some (Inguanzo et al., 2021; Kok et al., 2020; Zarkali et al., 2020) find no statistically significant abnormalities. In this situation a meta-analytic review can help explore robust patterns of altered GTA metrics in PD, to throw light on the pathophysiology and provide a framework for hypotheses in future studies.

The present study is the first quantitative meta-analysis of white matter global graph metrics in PD. Its purpose is to address the inconsistency in reports of the structural connectome in PD. The potential moderating effects of clinical and methodological factors are further addressed using subgroup analyses and meta-regression.

## Methods

### Search Strategy and Study Selection

A comprehensive search was performed for relevant studies in the PubMed, PsycINFO, Embase, and Web of Science databases up to August, 2021, following the Preferred Reporting Items for Systematic reviews and Meta-Analyses (PRISMA) guidelines (Moher et al., 2009). To find the largest pool of potentially eligible studies, the search strategy (detailed in Supplementary Table S2) included the 3 main themes of this systematic review: Parkinson's disease, GTA and dMRI. The reference lists of the retrieved studies and reviews were manually checked. Studies were considered eligible according to the following criteria: 1) comparing PD with HC; 2) using dMRI to investigate structural network alterations at the whole-brain (not sub-network) level; 3) reporting global topological parameters (including C_p_, L_p_, γ, λ, σ, E_loc_, E_glob_, network density, network strength, modularity) of the structural connectome (not regional/nodal measures); and 4) published in English in peer-reviewed original articles. To avoid sample overlap, among several papers using Parkinson Progression Markers Initiative (PPMI) databases (a multicenter observational study), the single study (Abbasi et al., 2018) with the largest sample size was included for meta-analysis. Studies were independently ascertained and checked by two researchers (C.Z. and X.S.), and inclusion and exclusion criteria were evaluated by consensus. There was almost perfect agreement (Cohen’s kappa = 0.841) (Viera & Garrett, 2005) between the two reviewers, any discrepancies (notably a study (Colon-Perez et al., 2018) disputed in the study inclusion session) being resolved by discussion or consulting a third senior investigator.

### Quality Appraisal

The quality and completeness of each included study were evaluated independently by two reviewers using a 13-point checklist (see Supplementary Table S3) adapted from previous meta-analyses on structural (Imms et al., 2019; Jiang et al., 2017) and functional (Pan et al., 2017) neuroimaging studies. The 13 points address three areas: the demographic and clinical aspects of participants (items 1–4), the methods for image acquisition and analysis (items 5–10), and the results and conclusions (items 11–13). For each item, 1, 0.5 or 0 scores were assigned when criteria were fully met, partially met or not met, respectively.

### Data Extraction

A data abstraction spreadsheet was created and data from eligible studies were extracted by one author (C.Z.) and double-checked by a second (X.S.). The following data were abstracted: family name of first author; publication year; demographic data of PD patients and HC; dMRI acquisition parameters; parcellation scheme; definition of edge, thresholds, basic measures of network topology (network density and network strength), measures of network segregation (C_p_, γ, E_loc_, and modularity) and network integration (L_p_, λ, and E_glob_), and small-worldness (σ); and clinical variables including illness duration, medication status, Unified Parkinson’s Disease Rating Scale (UPDRS) III scores, Mini-mental state examination (MMSE), Montreal Cognitive Assessment (MoCA), and Hoehn and Yahr (H&Y) stage. Corresponding authors were contacted via email if important data were not provided in the original report (Koirala et al., 2019; Shah et al., 2017; Zarkali et al., 2020). In two studies (Colon-Perez et al., 2018; Vriend et al., 2018) that did not report numerical data, this was extracted from graphical display using WebPlotDigitizer software (Rohatgi, 2020), a reliable and validated tool (Drevon et al., 2017). For a study (Kok et al., 2020) involving multiple independent PD and HC groups, each PD/HC pair was treated as a separate dataset. For studies (Colon-Perez et al., 2018; Galantucci et al., 2017; Wang et al., 2019, 2020; Wen et al., 2020) reporting results for multiple PD subgroups compared with one HC group, they were combined into a single group as recommended by the Cochrane collaboration (Higgins et al., 2021) (provided in Supplementary Material). For studies (Inguanzo et al., 2021; Wang et al., 2020; Wen et al., 2020) reporting the median and interquartile range rather than the first and third quartile, the mean was taken as equal to the median, and the standard deviation (SD) (if necessary) was calculated as recommended by Wan et al. (2014). In one study (Kamagata et al., 2017), the global network metrics calculated by probabilistic multi-shell multi-tissue constrained spherical deconvolution (CSD) were included in the analyses. For one study (Guan et al., 2019) with both binary and weighted networks, we extracted only weighted networks, as for the other included studies. In one study (Li et al., 2017) where data could not be extracted from a figure, we used the supplementary materials which reported results consistent with the main results, the only difference being a threshold number of streamlines (NOS) ≥ 5 rather than ≥ 3. Otherwise, if reported results were insufficient, the study was excluded from the meta-analysis (Shah et al., 2017).

### Data Analysis

We conducted all statistical analyses using Comprehensive Meta-Analysis software (version 3). For each global graph measure, the standardized mean difference between PD and HC across studies was calculated as Hedges’ g with a 95% confidence interval (CI). Hedges’ g and variance from each study were then pooled using a random-effects model to account for between-study heterogeneity (Borenstein et al., 2010). Being more conservative, the random-effects model yields a wider CI for the summary effect than the fixed-effect model and permits conclusions to be generalized to a wider range of situations (Borenstein et al., 2010). Pooled effect sizes were classified as small (0.2), medium (0.5) or large (0.8) (Cohen, 1988). Subgroup analyses were conducted for medicated (on-state) and medication-free (including both medication-naïve and off-state) patients (Suo et al., 2021c), tractography methods [probabilistic tractography (PT) and deterministic tractography (DT)], weights of the edge [fractional anisotropy (FA) and NOS], number of diffusion directions (≥ 30 and < 30), the definition of nodes [i.e., atlas: automated anatomic labeling (AAL) and non-AAL], and threshold approach (sparsity and absolute). A meta-regression was carried out to evaluate the potential moderating effects of clinical variables (mean age of the participants, percentage of males, mean duration of disease, mean UPDRS-III scores, and mean H&Y stages) which met the minimum requirement of meta-regression analysis (Borenstein et al., 2009; Higgins et al., 2021). To control Type I error, we employed the Knapp & Hartung adjustment (Viechtbauer et al., 2015).

Heterogeneity was assessed using the Q test, τ^2^ (tau-squared) and *I*^2^ values, which measure the true heterogeneity resulting from between-study variance rather than sampling error or chance. A statistically significant Q value (*P* < 0.10) shows that the true effects vary: τ^2^ is an absolute measure of heterogeneity, *I*^2^ a relative measure; *I*^2^ values of 25%, 50% and 75% indicate low, moderate and high proportions, respectively, of variance from the true heterogeneity (Higgins et al., 2003). To evaluate the impact of each study on the overall effect size and the stability of the results, a sensitivity analysis was performed by repeating the analysis after removing one study at a time (Gagne & Power, 2010). Potential publication bias was determined through visual inspection of funnel plot asymmetry (Sedgwick & Marston, 2015) and Egger’s linear regression test (Egger et al., 1997), and we used Duval and Tweedie’s ‘trim and fill’ method to adjust the impact of publication bias (Duval & Tweedie, 2000). Two-sided P-values < 0.05 were considered statistically significant.

## Results

### Search Results and Sample Characteristics

**Fig. 1:**
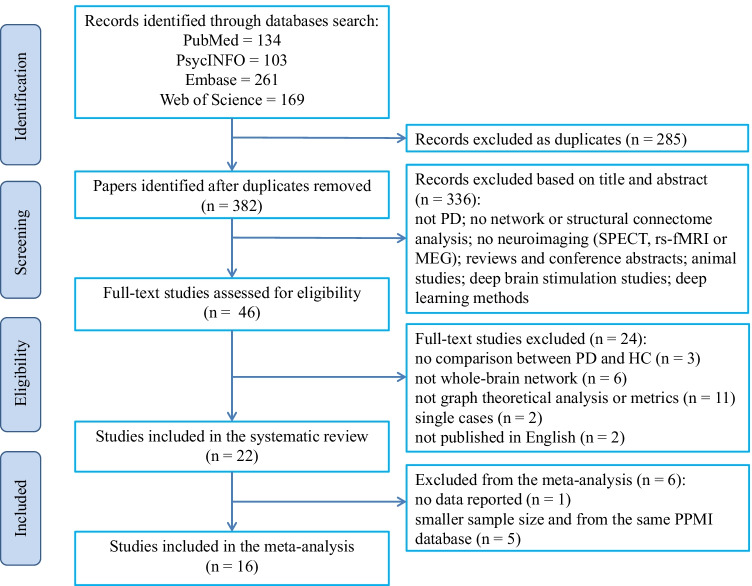
PRISMA flow diagram. The figure depicts the literature search and selection criteria. Abbreviations: HC, healthy controls; MEG, magnetoencephalography; PD, Parkinson’s disease; PPMI, Parkinson Progression Markers Initiative; rs-fMRI, resting-state functional magnetic resonance imaging; SPECT, single photon emission computed tomography

After removing 285 duplicate papers, 336 unrelated articles were rejected based on title and abstract, following which 46 full-text original articles were assessed for eligibility. Twenty-two studies met the inclusion criteria for systematic review. Of these, six studies could not be included in the meta-analysis: one did not report suitable data (Shah et al., 2017) and five reported data from the same PPMI database (Gou et al., 2018; Mishra et al., 2020; Wen et al., 2017a, b, 2018). Finally, sixteen whole-brain dMRI studies reporting on twenty-five datasets reporting graph theoretical measures were included in the meta-analysis (Fig. 1).

Demographic and clinical characteristics of the sixteen studies included in meta-analysis are provided in Table 1. These yielded aggregated data for 1476 participants: 916 PD patients and 560 HC. The mean age range for PD and HC samples were 57.9–69.4 years and 53.2–68.2 years, respectively. The PD sample (except for Colon-Perez et al. (2018), which did not report sex composition) consisted of 58.7% males, the HC sample 54.5% males. For the PD sample, mean UPDRS-III scores ranged from 14.4–37.2. Of the fifteen studies which gave information about medication status at the time of MRI scanning (one did not), four studies were of patients in the medication off-state, eight of the medication on-state, and three of medication-naïve patients. The diagnosis of PD was based on UK PD Society Brain Bank Clinical Diagnostic Criteria (Daniel & Lees, 1993; Hughes et al., 1992) in thirteen studies, Gelb-National Institute of Neurological Disorders and Stroke (NINDS) criteria (Gelb et al., 1999) in two studies, and was not specified in one study. The diagnosis of PD with mild cognitive impairment (MCI) in three studies (Galantucci et al., 2017; Inguanzo et al., 2021; Wang et al., 2020) included in the current meta-analysis was made according to level II of the Movement Disorder Society (MDS) proposed diagnostic criteria (e.g., at least 2 SDs below the normative scores for at least two cognitive tests within the five cognitive domains) (Litvan et al., 2012). The quality scores shown in Table 1 ranged from 9.5–12.5 (mean 11.4), a generally high quality. One of the lower scores among the 13 items was the clarity of the calculation of graph-theory metrics: most studies only introduced the concept of graph theory without giving the calculation formula or using multiple correction.

**Table 1:** Demographic and clinical characteristics of patients with Parkinson’s disease and healthy controls in 16 diffusion MRI studies (25 datasets)

Study	Subgroup	Sample size N (males)		Age (y)		Duration	UPDRS-III	H&Y stage	LEDD (mg/day)	MMSE^**^/ MoCA^***^	Medication status	Quality Score (/13)
		PD	HC	PD	HC							
Abbasi et al. (2018)		132 (84) ^a^	61 (38)	61.3	60.2	≤ 2 y	14.8	1 & 2	-	NA	Drug-Naïve	12.0
Colon-Perez et al. (2018)	PD-Well	31 (NA)	40 (33)	67.3	68.2	7.9 y	18.2	1–3	-	NA	On-State	9.5
	PD-MI	9 (NA)		69.4		6.2 y	15.7	1 3	-	NA		
Galantucci et al. (2017)	PD-NMCI	54 (29)	41 (15)	63	63	4.6 y	26.3	1.6	447	28.6^**^	On-State	11.5
	PD-MCI	54 (29)		64		6.2 y	37.2	2.1	691	27.6^**^		
Guan et al. (2019)		90 (50)	38 (16)	59.4	57.9	4.0 y	25.9	1.9	NA	27.0^**^	Off-State	12.0
Hu et al. (2020)	PD-ND	47 (25)	46 (22)	57.9	57.8	6.3 y	26.2	1.6	554	≥ 24^**^	On-State	11.5
	PD-D	20 (9)		58.1		5.4 y	27.7	1.4	501	≥ 24^**^		
Inguanzo et al. (2021)	PD-NMCI	35 (27)	51 (23)	63*	66*	7* y	15*	2.0	527*	NA	On-State	12.0
	PD-MCI	27 (19)		68*		8* y	15*	2.4	575*	NA		
Kamagata et al. (2017)		21 (12)	21 (8)	64.5	63.7	5.0 y	14.4	1.5	NA	NA	On-State	12.5
Koirala et al. (2019)		12 (4)	13 (8)	66.8	53.2	13.6 y	34.5	3.8	622	NA	On-state	11.0
Kok et al. (2020)		14 (10) ^b^	15 (10)	65	61.4	5.2 y	18.4	NA	NA	NA	Off-State	10.5
		19 (12) ^c^	18 (4)	60.7	56.9	4.8 y	25.4	NA	NA	NA		
Li et al. (2017)		35 (18)	26 (16)	61.3	62.8	3.5 y	30.0	2.1	NA	27.0^**^	Off-State	11.5
Nigro et al. (2016)		21 (15)	30 (20)	57.9	60.5	19.3 m	16.0	1.5	-	28.4^**^	Drug-Naïve	12.0
Vriend et al. (2018)		23 (17)	38 (20)	58.9	57.9	10 w	21.8	1.9	-	28.6^**^	Drug-Naïve	11.5
Wang et al. (2019)	PD-NDY	21 (14)	25 (17)	63.2	62.9	6.0 y	34.4	2.2	718	28.1^**^	Off-State	11.5
	PD-DY	21 (13)		60.3		9 y	35.7	2.5	745	27.9^**^		
Wang et al. (2020)	PD-NC	43 (23)	31 (16)	60.2	57	24* m	28.6	2.0*	NA	29^**, *^	NA	11.5
	PD-MCI	28 (13)		63.9		24* m	30.8	2.5*	NA	26^**, *^		
Wen et al. (2020)	PD-NA	28 (15)	32 (23)	60.4	62.1	5.3 y	17.5	2*	439	27.4^***^	On-State	12.0
	PD-A	31 (23)		62.7		6.8 y	25	2*	506	26.5^***^		
Zarkali et al. (2020)	PD-NVH	81 (47)	34 (16)	64.4	66.4	4.0 y	21.8	NA	457	28.9^**^	On-State	10.0
	PD-VH	19 (6)		64.6		4.8 y	29.2	NA	435	28.6^**^		

Results given as mean unless stated. Abbreviations: *H&Y* Hoehn and Yahr stage, *HC* healthy controls, *LEDD* levodopa equivalent daily dose, *MMSE* Mini-Mental State Examination, *MoCA* Montreal Cognitive Assessment, *PD* Parkinson’s disease, *PD-A* PD with apathy, *PD-D* PD with depression, *PD-DY* PD with dyskinesia, *PD-MCI* PD with mild cognitive impairment, *PD-MI* PD with memory impairment, *PD-NA* PD without apathy, *PD-NC* PD with normal cognition, *PD-ND* PD with no depression, *PD-NDY* PD with no dyskinesia, *PD-NMCI* PD without mild cognitive impairment, *PD-NVH* PD with no hallucinations, *PD-VH* PD with visual hallucinations, *PD-Well* PD without memory impairment, *UPDRS* Unified Parkinson's Disease Rating Scale

^*^median; ^**^Cognition was assessed using MMSE; ^***^Cognition was assessed using MoCA

^a^patients of the Parkinson Progression Markers Initiative (PPMI) database

^b^patients of the Dutch dataset (Data-NL)

^c^patients of the Canadian dataset (Data-CA); y, years; m, months; w, weeks; NA, not available

Data acquisition and GTA details are presented in Table 2. The number of acquisition diffusion directions was ≥ 30 in eleven studies and < 30 in three studies. Eight studies defined nodes through AAL and four studies by Desikan atlas. Four studies used sparsity threshold, ten used absolute threshold and two did not use a threshold approach. Six studies constructed a NOS-weighted brain structural network and five studies an FA-weighted network.

**Table 2:** Image acquisition parameters and network construction methods in the 16 diffusion MRI studies

Study	Parcellation scheme & number of nodes	Atlas regions removed	Tractography	Network framework weighting	Scanner B_0_ field	Number of directions	b-values	Threshold of fiber tracking	Threshold
Abbasi et al. (2018)	AAL 90	Cerebellum	DT	FA	3.0 T	64	1000 (NA)	FA ≥ 0.2, TA ≤ 45°	Sparsity, 0.1–0.3 (0.01)
Colon-Perez et al. (2018)	Freesurfer 82		DT	w(e_ij_)^a^	3.0 T	6/64	100/1000 (NA)	None	None
Galantucci et al. (2017)	Desikan (Freesurfer) 83		DT	FA	1.5 T	65	1000 (7 b0)	FA ≥ 0.15, TA ≤ 45°	Absolute, NOS ≥ 3, Edge in ≥ 40%^b^
Guan et al. (2019)	AAL 90	Cerebellum	DT	FA	3.0 T	32	1000 (NA)	FA ≥ 0.2, TA ≤ 45°	Sparsity, 0.1–0.3 (0.02)
Hu et al. (2020)	AAL 90	Cerebellum	DT	ROI-size-corrected fiber number	3.0 T	64	1000 (NA)	FA ≥ 0.2, TA ≤ 45°	Absolute, NOS ≥ 1,2,3,4,5, respectively
Inguanzo et al. (2021)	Desikan (Freesurfer) 86		PT	NOS	3.0 T	30	1000 (NA)	NA	Absolute, NOS ≥ 2, Edge in ≥ 50%^b^
Kamagata et al. (2017)	Desikan (Freesurfer) 84		PT (CSD)	NOS	3.0 T	32	1000/2000 (1 b0)	FOD ≥ 0.06, TA ≤ 45°	Sparsity 0.1–0.3 (0.05)
Koirala et al. (2019)	AAL 116		PT	NOS	3.0 T	32	1000 (5 b0)	NA	Absolute, Edge in ≥ 5%^b^
Kok et al. (2020)	Desikan (Freesurfer) 87	Ventricles, Cerebellum	PT (CSD)	FA	3.0 T^c^	60	4000 (7 b0)	FOD ≥ 0.1, TA ≤ 30°	Absolute, Edge in ≥ 50%^b^, Length 50–500 mm
					3.0 T^d^	32	700 (1 b0)		
Li et al. (2017)	AAL 90	Cerebellum	DT	FA	3.0 T	25	1000 (1 b0)	FA ≥ 0.2, TA ≤ 45°	Absolute, NOS ≥ 5
Nigro et al. (2016)	AAL 90	Cerebellum	DT	NOS × FA	3.0 T	27	1000 (1 b0)	FA ≥ 0.1, TA ≤ 35°	Absolute, NOS ≥ 3
Vriend et al. (2018)	BNA 210 and FSL FIRST 14		PT	NOS	3.0 T	30	1000 (5 b0)	5000 streamlines/voxel, curvature threshold 0.2	Sparsity, 0.1–0.2 (0.005); Edge in > 60%^b^
Wang et al. (2019)	AAL 90	Cerebellum	DT	NOS	3.0 T	30	1000 (1 b0)	FA ≥ 0.2, TA ≤ 45°	Absolute, NOS > 10
Wang et al. (2020)	AAL 90	Cerebellum	DT	NOS	3.0 T	25	1000 (1 b0)	FA ≥ 0.2, TA ≤ 45°	Absolute, NOS ≥ 3
Wen et al. (2020)	Destrieux 168		DT (QSDR)	QA	3.0 T	102	4000 (NA)	QA ≥ 0.02, TA ≤ 45°	Absolute, Length 30–450 mm
Zarkali et al. (2020)	Glasser 379		PT (CSD, ACT)	NOS × CSA	3.0 T	17/8/64	50/300/ 1000/2000	NA	None

Abbreviations: *AAL* automated anatomic labeling, *ACT* anatomically constrained tractography, *CSA* cross-sectional area, *CSD* constrained spherical deconvolution, *DT* deterministic tractography, *FA* fractional anisotropy, *FOD* fiber orientation distribution, *FSL* functional MRI of the brain (FMRIB) software library, *NOS* number of streamlines, *PT* probabilistic tractography, *QA* quantitative anisotropy, *QSDR* q-space diffeomorphic reconstruction, *TA* turning angle

^a^Calculation of network-weighted edges described by Colon-Perez et al. (2018)

^b^Connection from node i to node j considered to exist if present in > N% of participants

^c^image acquisition of Dutch dataset (Data-NL)

^d^image acquisition of Canadian dataset (Data-CA), *NA* not available

Table 3 summarizes the main findings of the sixteen articles in the meta-analysis. Of twenty datasets reporting C_p_, five datasets reported a decrease and fifteen no significant change between PD (or PD subgroup) and HC. Of twenty datasets reporting L_p_, eight reported an increase, two a decrease and ten no significant change. Of eighteen datasets reporting E_glob_, ten reported a decrease and eight no significant change.

**Table 3:** Alterations of graph metrics in the 16 diffusion MRI studies of patients with Parkinson’s disease *vs* healthy controls

Study	Subgroups	C_p_	L_p_	γ	λ	σ	E_loc_	E_glob_	Density	Strength	Modularity
Abbasi et al. (2018)	None	↓	↑	…	…	…	…	↓	…	…	…
Colon-Perez et al. (2018)	PD-Well	-	-	…	…	-	…	…	-	↓	…
	PD-MI	-	↓	…	…	-	…	…	-	↓	…
Galantucci et al. (2017)	PD-NMCI	-	-	…	…	-	…	-	-	…	…
	PD-MCI	↓	↑	…	…	-	…	↓	↓	…	…
	PD-MCI *vs* PD-NMCI ^a^	↓	-	…	…	-	…	↓	-	…	…
Guan et al. (2019)	None	-	-	…	…	…	-	-	…	…	…
Hu et al. (2020)	PD-D	-	↑	-	↑	↑	↑	↓	…	-	…
	PD-ND	-	-	↓	-	-	↓	-	…	-	…
	PD-D *vs* PD-ND ^a^	-	-	↑	-	↑	↑	-	…	-	…
Inguanzo et al. (2021)	PD-NMCI	…	…	-	-	-	…	…	…	…	-
	PD-MCI	…	…	-	-	-	…	…	…	…	-
Kamagata et al. (2017)	None	↓	↑	…	…	↓	…	↓	…	↓	…
Koirala et al. (2019)	None	…	↓	…	…	…	…	…	…	…	…
Kok et al. (2020)	Data NL ^b^	…	…	…	…	…	-	-	…	…	…
	Data CA ^c^	…	…	…	…	…	-	-	…	…	…
Li et al. (2017)	None	-	↑	-	-	-	-	↓	…	…	…
Nigro et al. (2016)	None	↓	-	…	…	…	…	↓	-	↓	…
Vriend et al. (2018)	None	↓	…	…	…	…	…	↓	…	…	↑
Wang et al. (2019)	PD-NDY	-	↑	…	…	-	-	↓	…	…	…
	PD-DY	-	-	…	…	-	-	-	…	…	…
	PD-DY *vs* PD-NDY ^a^	↑	↓	…	…	-	↑	↑	…	…	…
Wang et al. (2020)	PD-NC	-	-	-	-	-	-	-	…	…	…
	PD-MCI	-	↑	↑	-	↑	-	↓	…	…	…
	PD-MCI vs PD-NC ^a^	-	-	-	-	-	-	-	…	…	…
Wen et al. (2020)	PD-NA	-	-	…	…	…	-	-	…	…	…
	PD-A	-	↑	…	…	…	-	↓	…	…	…
	PD-A *vs* PD-NA ^a^	-	↑	…	…	…	-	↓	…	…	…
Zarkali et al. (2020)	PD-NVH	-	-	…	…	…	…	…	…	…	-
	PD-VH	-	-	…	…	…	…	…	…	…	-

Abbreviations: *↑/↓* graph metrics in PD patients were significantly higher/lower, respectively, than in HC, *-* no significant difference between PD and HC, *…* metric was not reported, *PD-A* PD patients with apathy, *PD-D* PD patients with depression, *PD-DY* PD patients with dyskinesia, *PD-MCI* PD patients with mild cognitive impairment, *PD-MI* PD patients with memory impairment, *PD-NA* PD patients with no apathy, *PD-NC* PD patients with normal cognition, *PD-ND* PD patients with no depression, *PD-NDY* PD patients with no dyskinesia, *PD-NMCI* PD patients without mild cognitive impairment, *PD-NVH* PD patients with no hallucinations, *PD-VH* PD patients with visual hallucinations, *PD-Well* PD patients without memory impairment, *C*_*p*_ clustering coefficient, *E*_*glob*_ global efficiency, *E*_*loc*_ local efficiency, *L*_*p*_ characteristic path length, *γ* normalized clustering coefficient, *λ* normalized characteristic path length, *σ* small-worldness

^a^↑/↓, the former subgroup higher/lower compared with the latter subgroup

^b^graph metrics of the Dutch dataset (Data-NL)

^c^graph metrics of the Canadian dataset (Data-CA)

### Meta-analysis and Meta-Regression analysis

**Fig. 2:**
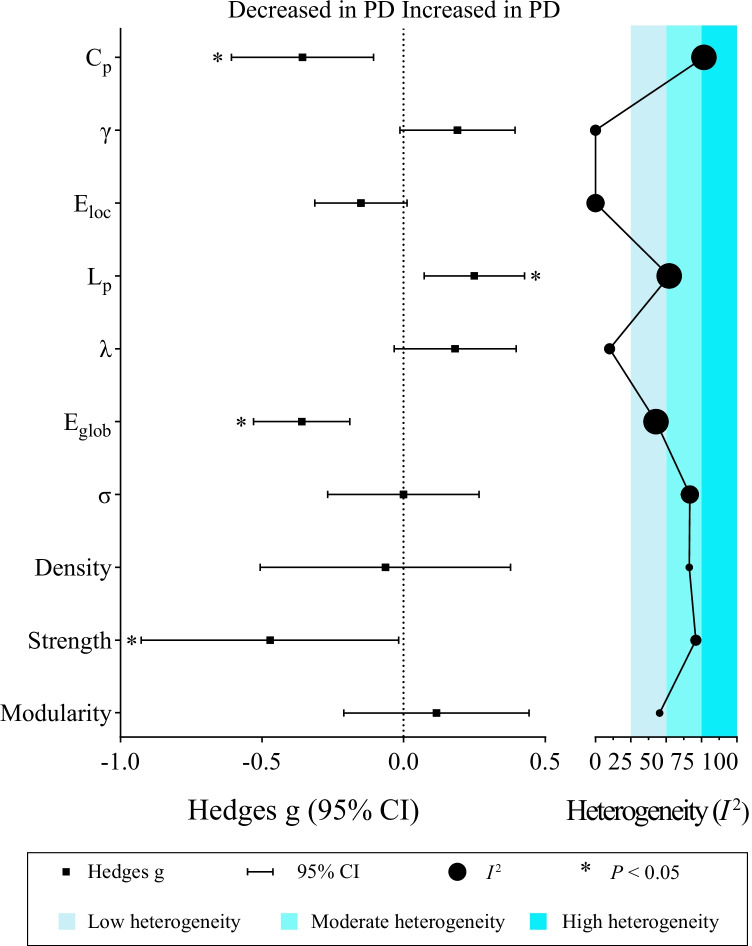
Results of the meta-analyses. For each topological property named on the vertical axis, the figure shows pooled effect sizes as Hedges’ g (with 95% CI bars) in the left panel, and heterogeneity values (*I*^2^) in the right panel (bands are color-coded as shown in the legend); the size of the circles in the latter represents number of studies (3, 4, 8 and 13 from smallest to largest, respectively). The asterisks denote statistically significant effect size. Abbreviations: C_p_, clustering coefficient; E_glob_, global efficiency; E_loc_, local efficiency; L_p_, characteristic path length; γ, normalized clustering coefficient; λ, normalized characteristic path length; σ, small-worldness

The results of the main meta-analysis are summarized in Fig. 2 and Table 4, the subgroup analyses in Supplementary Table S4 and the meta-regression analysis in Supplementary Table S5. Details of these analyses are presented below, grouped by the main category of network measurements.

**Table 4:** Meta-analysis effect size, heterogeneity and publication bias in the 16 diffusion MRI studies of patients with Parkinson’s disease *vs* healthy controls

Network topology measure	Number of studies reporting	Number of patients (N)	Hedges’ g			Z	P	Heterogeneity				Egger test
			g	Lower g	Upper g			τ^2^	*I*^2^ (%)	Q	P	P
**Network segregation**												
C_p_	13	1272	-0.357	-0.608	-0.106	-2.789	0.005*	0.159	76.886	51.915	< 0.001**	0.038*
γ	4	389	0.190	-0.013	0.394	1.831	0.067	< 0.001	< 0.001	0.994	0.803	NA
E_loc_	8	628	-0.151	-0.314	0.012	-1.811	0.070	< 0.001	< 0.001	4.265	0.749	0.055
Modularity	3	308	0.116	-0.211	0.443	0.694	0.488	0.038	45.429	3.665	0.160	NA
**Network integration**												
L_p_	13	1236	0.250	0.073	0.427	2.766	0.006*	0.053	52.217	25.113	0.014**	0.332
λ	4	389	0.182	-0.033	0.398	1.656	0.098	0.005	10.106	3.337	0.342	NA
E_glob_	13	1124	-0.359	-0.529	-0.190	-4.155	< 0.001*	0.040	42.804	20.981	0.051**	0.851
**Small-worldness**												
σ	8	727	0.0004	-0.268	0.267	-0.003	0.998	0.098	66.889	21.141	0.004**	0.193
**Basic measures**												
Network density	3	280	-0.064	-0.506	0.378	-0.284	0.776	0.101	66.469	5.965	0.051**	NA
Network strength	4	286	-0.471	-0.926	-0.017	-2.032	0.042*	0.150	71.108	10.383	0.016**	NA

Abbreviations: *C*_*p*_ clustering coefficient, *E*_*glob*_ global efficiency, *E*_*loc*_ local efficiency, *L*_*p*_ characteristic path length, *γ* normalized clustering coefficient, *λ* normalized characteristic path length, *σ* small-worldness (= γ/ λ)

*significant at *P* < 0.05 for effect size and Egger test; **significant at *P* < 0.10 for heterogeneity

#### Information Segregation Measures

Thirteen studies (N_PD_ = 809, N_HC_ = 463) reported clustering coefficient C_p_ (Abbasi et al., 2018; Colon-Perez et al., 2018; Galantucci et al., 2017; Guan et al., 2019; Hu et al., 2020; Kamagata et al., 2017; Li et al., 2017; Nigro et al., 2016; Vriend et al., 2018; Wang et al., 2019, 2020; Wen et al., 2020; Zarkali et al., 2020). The main meta-analysis showed significantly decreased C_p_ in PD patients compared to HC (g = -0.357, 95% CI: -0.608 to -0.106, *P* = 0.005). However, this effect had high heterogeneity (*I*^*2*^ = 76.9%, Q [12] = 51.915, *P* < 0.001, τ^2^ = 0.159) and evidence of publication bias (P = 0.038, Supplementary Fig. S1A). Duval and Tweedie’s ‘trim and fill’ yielded two potentially missing studies on the left side of the plot, lowering Hedges’ g to − 0.456 (95% CI: -0.712 to -0.200). In the medication status subgroup analysis of C_p_ (Supplementary Table S4), the effect remained significant for on-state and drug-naïve/off-state subgroups and the impact of heterogeneity was reduced in the on-state subgroup (*I*^*2*^ = 36.0%). Over the twelve studies in which it was possible, meta-regression analysis for C_p_ (Supplementary Table S5) found a significant association between sex (male %) and Hedges’ g [regression coefficient (β) = − 5.217, *P* = 0.014]. However, UPDRS-III scores, age, H&Y stages or duration had no significant moderating effect on the combined effect size (all *P* > 0.05).

Four studies (N_PD_ = 235, N_HC_ = 154) reported normalized clustering coefficient γ (Hu et al., 2020; Inguanzo et al., 2021; Li et al., 2017; Wang et al., 2020). The meta-analysis showed no significant difference in γ of PD compared to HC (g = 0.190, 95% CI: -0.013 to 0.394, *P* = 0.067) with low heterogeneity (Q [3] = 0.994, *P* = 0.803, *I*^*2*^ < 0.001%, τ^2^ < 0.001).

Three studies (N_PD_ = 185, N_HC_ = 123) reported modularity (Inguanzo et al., 2021; Vriend et al., 2018; Zarkali et al., 2020). The meta-analysis showed no significant difference in modularity of PD compared to HC (g = 0.116, 95% CI: -0.211 to 0.443, *P* = 0.488) with low heterogeneity (Q [2] = 3.665, *P* = 0.160, *I*^*2*^ = 45.4%, τ^2^ = 0.038). The small number of included studies for γ and modularity precluded analysis of publication bias, subgroup analysis and meta-regression.

Eight datasets from seven studies (N_PD_ = 397, N_HC_ = 231) reported local efficiency E_loc_ (Guan et al., 2019; Hu et al., 2020; Kok et al., 2020; Li et al., 2017; Wang et al., 2019, 2020; Wen et al., 2020). The main meta-analysis showed no significant difference in E_loc_ (g = -0.151, 95% CI: -0.314 to 0.012, P = 0.070) with low heterogeneity (Q [7] = 4.265, P = 0.749, *I*^*2*^ < 0.001%, τ^2^ < 0.001) and no evidence of publication bias (P = 0.055, Supplementary Fig. S1B). In the medication status subgroup analysis, there was no significant effect.

#### Information Integration Measures

Thirteen studies (N_PD_ = 798, N_HC_ = 438) reported characteristic path length L_p_ (Abbasi et al., 2018; Colon-Perez et al., 2018; Galantucci et al., 2017; Guan et al., 2019; Hu et al., 2020; Kamagata et al., 2017; Koirala et al., 2019; Li et al., 2017; Nigro et al., 2016; Wang et al., 2019, 2020; Wen et al., 2020; Zarkali et al., 2020). The main meta-analysis showed significantly increased L_p_ of PD compared to HC (g = 0.250, 95% CI: 0.073 to 0.427, *P* = 0.006). This effect had moderate heterogeneity (Q [12] = 25.113, *P* = 0.014, *I*^*2*^ = 52.2%, τ^2^ = 0.053), with no evidence of publication bias (*P* = 0.332, Supplementary Fig. S1C). Nevertheless, ‘trim and fill’ yielded 2 potentially missing studies on the left side of the plot, lowering Hedges’ g to 0.200 (95% CI: 0.027 to 0.373). For the drug-native/off-state subgroup, the significance of the increased L_p_ in PD patients was retained (k = 5, *P* < 0.001) with no significant heterogeneity. However, the on-state PD subgroup showed no significant difference in L_p_ from HC (k = 7, *P* = 0.424, *I*^*2*^ = 71.7%). For the meta-regression of L_p_, an outlier analysis required the exclusion of a study (Koirala et al., 2019) whose duration, H & Y stages and sex ratio were not in the range of the mean ± 2 SDs. After that, only age had a negative moderating tendency (k = 12, β = -0.056, 95% CI: -0.113 to 0.0002, R^2^ = 0.714, *P* = 0.051) on the combined effect size.

Four studies (N_PD_ = 235, N_HC_ = 154) reported normalized characteristic path length λ (Hu et al., 2020; Inguanzo et al., 2021; Li et al., 2017; Wang et al., 2020). The main meta-analysis showed no significant difference in λ between PD and HC (g = 0.182, 95% CI: -0.033 to 0.398, P = 0.098), with low heterogeneity (Q [3] = 3.337, P = 0.342, *I*^2^ = 10.1%, τ^2^ = 0.005).

Thirteen datasets from twelve studies (N_PD_ = 702, N_HC_ = 422) reported global efficiency E_glob_ (Abbasi et al., 2018; Galantucci et al., 2017; Guan et al., 2019; Hu et al., 2020; Kamagata et al., 2017; Kok et al., 2020; Li et al., 2017; Nigro et al., 2016; Vriend et al., 2018; Wang et al., 2019, 2020; Wen et al., 2020). In the main meta-analysis the combined effect size was small but statistically significant (g = -0.359, 95% CI: -0.529 to -0.190, *P* < 0.001), with low heterogeneity (Q [12] = 20.981, *P* = 0.051, *I*^*2*^ = 42.804%, τ^2^ = 0.040) and no evidence of publication bias (*P* = 0.851, Supplementary Fig. S1D). As with C_p_, the effect remained significant in the drug-naïve/off-state subgroup, and there was no significant heterogeneity in the on-state. Meta-regression analysis of E_glob_ revealed no effect of potential moderators e.g. UPDRS-III scores, H&Y stages, duration, age or sex (all P > 0.05).

#### Small-worldness Measures

Eight studies (N_PD_ = 446, N_HC_ = 281) reported the small-worldness parameter σ (Colon-Perez et al., 2018; Galantucci et al., 2017; Hu et al., 2020; Inguanzo et al., 2021; Kamagata et al., 2017; Li et al., 2017; Wang et al., 2019, 2020); meta-analysis revealed no significant difference in σ between PD and HC (g = -0.0004, 95% CI: -0.268 to -0.267, *P* = 0.998), with moderate heterogeneity (Q [7] = 21.141, *P* = 0.004, *I*^*2*^ = 66.889%, τ^2^ = 0.098) and no evidence of publication bias (*P* = 0.193, Supplementary Fig S1E). The effect sizes for the on-state and off-state PD subgroups were also not significant compared to HC.

#### Basic Network Measures

Three studies (N_PD_ = 169, N_HC_ = 111) reported network density (Colon-Perez et al., 2018; Galantucci et al., 2017; Nigro et al., 2016); the meta-analysis showed no significant difference of network density between PD and HC (g = -0.064, 95% CI: -0.506 to 0.378, *P* = 0.776) with moderate heterogeneity (Q [2] = 5.965, *P* = 0.051, *I*^*2*^ = 66.5%, τ^2^ = 0.101). Four studies (N_PD_ = 149, N_HC_ = 137) reported network strength (Colon-Perez et al., 2018; Hu et al., 2020; Kamagata et al., 2017; Nigro et al., 2016); the meta-analysis showed a significant decrease in PD compared to HC (g = -0.471, 95% CI: -0.926 to -0.017, *P* = 0.042), with high heterogeneity (Q [4] = 10.383, *P* = 0.016, *I*^*2*^ = 71.1%, τ^2^ = 0.150). The small number of included studies for network density and network strength precluded analysis of publication bias, subgroup analysis and meta-regression.

#### Subgroup Analyses by Methodological Factors

The significantly decreased clustering coefficient C_p_ in PD compared to HC was retained in subgroups for the PT method, the FA-weighted network, diffusion gradient directions ≥ 30, non-AAL atlas and sparsity threshold, but not for the DT method, the NOS-weighted network, directions < 30, AAL atlas and absolute threshold (Supplementary Table S4). The significantly increased characteristic path length L_p_ in PD compared to HC was retained for the DT method, AAL atlas and the FA-weighted network but not for the PT method, non-AAL atlas and the NOS-weighted network. The significantly decreased local efficiency E_loc_ in PD compared to HC was found for the DT method and directions ≥ 30. The significantly decreased global efficiency E_glob_ in PD compared to HC was retained for the DT method, the NOS-weighted network and diffusion gradient directions ≥ 30 but not for the PT method, the FA-weighted network, or directions < 30.

#### Sensitivity Analyses

Sensitivity analysis indicated that individual study or datasets could affect the statistically significant difference in E_loc_ [when Data-CA (the Canadian dataset) of Kok et al. (2020) was removed], γ (when Hu et al. (2020) was removed), λ (when Inguanzo et al. (2021) was removed) and network strength (when Colon-Perez et al. (2018), Kamagata et al. (2017) or Nigro et al. (2016) was removed) between PD and HC (for details, see Supplementary Fig. S2). In contrast, no individual study significantly affected the difference between PD patients and HC in C_p_, L_p_, E_glob_, σ, network density or modularity.

## Discussion

To the best of our knowledge, this is the first meta-analysis assessing the consistency of brain structural topological properties in PD based on dMRI studies using GTA. We found a significant decrease in C_p_, E_glob_, and network strength of the structural connectome in PD, and a significant increase in L_p_. In contrast, E_loc_, γ, λ, σ, density and modularity showed no significant alteration in the structural connectome in PD. In subgroup analyses, the statistical difference of L_p_ between PD and HC was maintained in the drug-naïve/off-state patients but lost in the on-state patients. Meta-regression analysis revealed that sex (male %) was a confounder of C_p_ in the meta-analysis, and age had a negative moderating tendency on L_p_.

We discuss the pathophysiological significance of these results below, but it is useful first to outline their basic network-theory interpretation. In general, decreased clustering coefficient C_p_ implies decreased information segregation, and decreased global efficiency E_glob_ and increased characteristic path length L_p_ both imply decreased network integration. There were no significant effects on γ, E_loc_ or modularity (also measures of information segregation) or λ (also a measure of integration) or on network density, but for four of these the number of studies was very low. A decrease in both network segregation and integration is characteristic of a ‘weaker small-worldization’ pattern (Suo et al., 2018). The small-worldness parameter σ is the ratio of the normalized clustering coefficient to the normalized characteristic path length: as the absolute clustering coefficient C_p_ is decreased and the absolute characteristic path length L_p_ is increased, one would expect σ to be decreased. However, the parameters (γ, λ and σ) might be affected by the normalization processes of C_p_ and L_p_. Specifically, γ or λ is normalized relative to C_p_ or L_p_ of matched random networks that preserve the number of nodes and edges of the real network. The results of comparing network properties between groups may differ from the results of normalized network properties due to the different degree of distribution of matched random networks across individuals. Such seemingly contradictory results are also reported in patients with focal epilepsy (increased γ and decreased C_p_) (Výtvarová et al., 2017), and patients with Alzheimer's disease (decreased λ and increased L_p_) (Stam et al., 2009).

### Weaker Small-Worldization

C_p_ is an important global measure of network segregation, quantified as the ratio of the number of connections that exist between the direct neighbors of a node to the maximum number of possible connections, averaged over the network (Watts & Strogatz, 1998). The decreased C_p_ implies poorer network segregation resulting in less efficient information processing at the local level in functionally specific areas, and such a network is less robust to node failure (Bullmore & Sporns, 2012; Rubinov & Sporns, 2010). It has been reported that PD-MCI showed decreased C_p_ compared to PD without MCI (Galantucci et al., 2017). Our meta-regression analysis suggests that decreased C_p_ was associated with the higher percentage of men with PD. Consistent with this, male PD patients reportedly show faster progression of daily living difficulties and cognitive decline (Bakeberg et al., 2021; Iwaki et al., 2021), and faster development of impaired brain structural impairment by structural MRI and GTA (Yadav et al., 2016). The reasons for the high heterogeneity are not fully understood, but methodological factors and the heterogeneity of PD sample no doubt contribute. These factors will be discussed in more detail below.

For network integration, we considered three measures: L_p_, λ and E_glob_. Integration is a crucial feature of an efficient network architecture, allowing for rapid communication of information across distributed regions (Sporns, 2013). The findings of decreased E_glob_ and increased L_p_ in PD relative to HC indicate disruption of global network integration. In the medication subgroup analysis, the effect of increased L_p_ remained significant in the drug-naïve/off-state patients while effect size for on-state patients was not significant. These results may be associated with the ‘normalization’ effect of dopaminergic medication in PD, as levodopa tends to normalize the connectivity of the striato-thalamo-cortical motor circuits and default mode network, and the disrupted network topology (Berman et al., 2016; Gao et al., 2017; Zhong et al., 2019). Although medication was discontinued at least 12 h before MRI scanning, we cannot completely discount potential confounding chronic effects of dopaminergic drugs. Our meta-regression results suggest that older patients had a lower propensity toward L_p_ in structural network, probably due to a reorganization of brain structural connectome in aging. This is supported by a study reporting decrease in L_p_ with age that included participants of similar age (average at baseline 63.5 years, at follow-up 68.0 years) to the PD patients in this meta-analysis (Coelho et al., 2021). In one study disease duration was positively correlated with L_p_ and negatively correlated with E_glob_ (Li et al., 2017), although the corresponding meta-regressions in the current study did not reveal disease duration to have any significant moderating role to influence network properties. In various studies of PD patients L_p_ was negatively associated with working memory (Colon-Perez et al., 2018) and dyskinesia (Wang et al., 2019), and positively associated with UPDRS-III scores (Colon-Perez et al., 2018); also E_glob_ was negatively related to motor symptoms (Kok et al., 2020) and lower in PD patients with MCI compared to those without MCI (Galantucci et al., 2017), and in MCI compared to HC (Berlot et al., 2016).

In formal terms, the brain’s small-world organization strikes an optimal balance between segregation (reflected by C_p_, γ, E_loc_ and modularity) and integration (reflected by L_p_, λ and E_glob_) of information processing. This organization supports efficient integration and specialized information processing at low connection cost (Liao et al., 2017; Telesford et al., 2011). The small-worldness σ is the ratio of γ to λ, and reflects the network showing higher clustering and similar path lengths to a network connected by randomly assigned edges (Watts & Strogatz, 1998). From the perspectives of segregation and integration, altered small-world properties in disease fall into four patterns: regularization, randomization, and stronger and weaker small-worldization (Suo et al., 2018). We found significant reduction in C_p_ and E_glob_, and increase in L_p_ of PD relative to HC, which represents lower network segregation and integration and indicate weaker small-worldization of the structural connectome, although the change did not reach statistical significance in σ. Notably, σ in PD patients was significantly lower using PT when compared to HC, whereas there was no difference in σ calculated by DT (Kamagata et al., 2017). Additionally, increased σ might be related to depression in PD (Hu et al., 2020). Although no significant outcomes were obtained for other metrics, the P-values for overall effects of γ, λ and E_loc_ were less than 0.10. Note that leave-one-out sensitivity analysis can yield differing results when specific studies are removed. That the E_loc_ of PD *vs* HC is significantly decreased when the Data-CA set of Kok et al. (2020) is removed may be because the small size of the Data-CA set (19 PD patients and 18 HC) makes it vulnerable to sampling error (Lin, 2018), or because the lower b-values (700) and limited number of dMRI diffusion directions were sub-optimal for CSD tractography; in any case the effects are unsurprisingly exposed when only 4 studies are included in the pooled effect estimates for γ, λ and network strength. Caution is therefore needed, until additional data can be analyzed and reported.

### Diagnostic Performance and Mechanistic Insight with Graph Theoretical Analysis

Given the many reports of altered GTA parameters in PD, the question of their diagnostic power has received attention. Some studies have applied support vector machine to GTA metrics and matrices to assess their classification performance (Kamagata et al., 2017; Kazeminejad et al., 2017; Suo et al., 2021a, b). PD patients could be distinguished from HC with 78% accuracy by combining five global metrics (C_p_, L_p_, E_glob_, σ and network strength); probabilistic multi-shell, multi-tissue CSD tracking performed better than deterministic and probabilistic single-shell, single-tissue CSD tracking (Kamagata et al., 2017). GTA metrics could differentiate between early-stage PD patients and HC with 73% balanced accuracy (Suo et al., 2021b). A functional study applied GTA to rs-fMRI to distinguish PD patients from HC with accuracy of ~ 95% in a leave-one-out cross-validation test (Kazeminejad et al., 2017). These studies show the limited ability of structural measures to identify PD patients, especially given the variability in the findings and the additional cost (financial, expert time, infrastructure) necessary to obtain these metrics. In particular, a study revealed that classification accuracy can be improved by multiple kernel support vector machine combining GTA metrics with original functional connections (Chen et al., 2020). This suggests that GTA metrics in combination with other neuroimaging measures may help differentiate patients. However, there is a long way to go before their clinical application in the PD structural connectome.

From the mechanistic perspective, a critical pathological feature of PD is the deposition of fibrillary aggregates consisting mainly of α-synuclein within Lewy bodies and Lewy neurites (Spillantini et al., 1998). Pathological accumulation of α-synuclein can alter synaptic and structural plasticity by reducing the activity of N-methyl-D-aspartate receptors, leading to further disruption of synaptic and axonal connections (Bellucci et al., 2016; Braak & Del Tredici, 2008). Consistent with this, we found reduced network strength in PD patients in the dMRI structural network. The implication is that white matter disconnections in the PD structural connectome can impair efficient information exchange, resulting in reduced network computational efficiency (lower segregation and integration). This might not be seen in relatively early disease, where synaptic dysfunction leads to minor axonal loss, while structural connectivity may not be significantly altered.

### Methodological Considerations

Many methodological factors may have influenced the overall effect sizes in this meta-analysis: parcellation schemes, the definition of edges, threshold, and fiber tracking technology (shown in Table 2). We consider these in turn.

The commonest brain parcellation methods used the AAL and Desikan atlas to define the network nodes. None of the available parcellation schemes optimally addresses all challenges (Arslan et al., 2018). Since the number of nodes (82–379) in the included studies is on a similar scale, parcellation has less influence on the results of the network parameters and allows for cross-study comparisons (Zalesky et al., 2010). GTA studies have assessed abnormalities at both the global level and the nodal (brain area) level. However, because of the complexity and variety of parcellation schemes, we could find no robust way to conduct a quantitative meta-analysis at the nodal level. Our results are therefore only at the whole-brain level. AAL atlas (8 studies) was the commonest parcellation method in the included studies; however, most did not report the detailed value of the nodal parameters. The most commonly altered brain areas were in basal ganglia, sensorimotor and orbitofrontal areas, which have been related to the motor and nonmotor symptoms of PD (Kobayakawa et al., 2017; Neumann et al., 2018; Tessitore et al., 2014).

Although the networks constructed in the included studies were all of the weighted type, the definitions of the edges were diverse, and this is known to affect network efficiency estimates (Zhong et al., 2015). Our subgroup analysis found differences not only in E_glob_, but also in C_p_ and L_p_ between differently-weighted methods. Therefore, the heterogeneity we observed across studies might be, in part, due to differences of edge definition. These methodological issues are not fully solved, which hampers cross-study comparisons of network topology.

Data noise and algorithm errors mean that the raw individual networks are likely to contain spurious connections, and the purpose of thresholding is to remove edges with very small weights that are not physically credible (Hagmann et al., 2007). There are two approaches: absolute threshold values and sparsity threshold values. In the former approach only edges that exceed a certain statistical significance (or some other criterion) are retained: e.g., retaining only connections with NOS ≥ α (α is a critical value selected by the investigator). However, it will retain a different number of edges among different individuals, leading to biased network properties. In the latter approach sparsity threshold values are calculated as the ratio of the number of actual connections divided by the maximum possible number of connections in the network, which normalizes each individual network to the same number of nodes and edges. Multiple studies indicate that most network attributes are dependent on the sparsity (De Reus & van den Heuvel, 2013; Fornito et al., 2013; van Wijk et al., 2010), so network comparison is still biased by the arbitrary choice of the threshold or range of thresholds (Cheng et al., 2012a, b). In particular, our subgroup analysis showed significantly decreased C_p_ of PD compared to HC using sparsity threshold, but showed no significant difference using an absolute threshold. This seems to suggest that sparsity threshold may be more sensitive than the absolute threshold to differences in C_p_ of PD related to HC, but this inference must be treated with caution because of the limited number of included articles.

Tractography including DT and PT is required to determine if two nodes are anatomically connected in a structural network based on dMRI. While DT is most widely used, it has a limited capacity for resolving crossing fibers. PT characterizes the uncertainty in the local fiber orientation estimates, and this is theoretically better than DT in respect of inter-individual variability of the tracked streamlines, and fiber-crossing issues (Jeurissen et al., 2019; Zalesky et al., 2016). In fact, one study found PT more sensitive than DT in detecting disruptions in structural connections in PD (Kamagata et al., 2017). In our study statistically significant differences were detected in more network metrics in the DT than PT subgroup, although this result should be interpreted with caution until more primary studies are published.

Overall, our methodological subgroup analysis reflects the absence of a methodological gold standard. Interestingly, E_glob,_ E_loc_ and C_p_ maintained statistical differences with ≥ 30 diffusion gradient directions, but these were lost with < 30 directions. This probably reflects the fact that abnormalities in graph theoretical parameters are better identified using a higher number of dMRI directions. Again, because of the low number of studies in each subgroup, the results of our analysis should be considered exploratory. Future studies should perform subgroup analysis to further confirm the impact of different parcellation schemes, definition of edges, threshold, and fiber tracking technology.

### Limitations

PD patients are clinically heterogeneous. We did not focus on a particular subgroup but included patients ranging from a de novo state to manifest PD patients with different motor subtypes including dyskinesia, tremor-dominant and postural instability and gait difficulty subtypes, as well as patients with specific non-motor symptoms, e.g., MCI, depression, hyposmia, apathy, and visual hallucinations. Although the included studies (Galantucci et al., 2017; Inguanzo et al., 2021; Wang et al., 2020) all evaluated MCI using MDS Task Force level II criteria (Litvan et al., 2012), the different scales used for this criterion (the cut-off scores are taken as 2 SD in Galantucci et al. (2017) and 1.5 SD in Wang et al. (2020) below normative means) may have caused inconsistencies among the included MCI patients. Additionally, there is substantial heterogeneity in cognitive subdomain deficits in PD-MCI. Full study of the different subtype profiles will require a stratified statistical analysis which is beyond the scope of the current study, although it is a focus of ongoing work. This approach allowed us to focus on the commonality between patients, rather than the differences between subgroups. In any case, the number of subtype studies did not meet the minimum requirement of subgroup analyses. As studies proliferate, it will be important to investigate global topology alterations in different subtypes of PD.

The heterogeneity of medication status might have influenced the results; for example, levodopa can alter global and local efficiency measures of the functional connectivity network in PD (Berman et al., 2016). We performed subgroup analyses to investigate this, but a tightly controlled study will be required to explore the effect of levodopa on structural topological properties in PD. There were too few studies to allow us to control for other confounding factors (such as clinical, MRI acquisition and network construction methods). Finally, the Egger test indicated a potential publication bias in the C_p_ analysis. Thus, it will be important to validate our findings by an updated meta-analysis.

The small number of studies included in some subgroup analyses (e.g., tractography methods) is a significant limitation. There is no clear consensus for how many studies are needed for the meta-analysis (Greco et al., 2013; Müller et al., 2018; Pigott, 2012; Valentine et al., 2010): some view 17–20 studies are desirable (Eickhoff et al., 2016), while others argue that meta-analysis can be conducted with as few as 2 studies which meet quality standards and statistical requirements (Pigott, 2012; Valentine et al., 2010). Müller et al. (2018) stated that required number of experiments of a meta-analysis is strongly dependent on the expected effect size. Finally, Valentine et al. (2010) showed that meta-analysis is a better choice for synthesis than alternatives which are typically based on less tenable assumptions and less transparent processes. We have therefore chosen to proceed by meta-analysis, recognizing that results need to be interpreted with caution because of limited statistical power; we suggest that our fundings are best used in hypothesis generation to underpin future research.

### Future Directions

Most of these studies constructed the structural connectome using FA to define the edges. Future studies might usefully explore other diffusivity parameters (mean diffusivity, and axial and radial diffusivity) to provide a more comprehensive picture of the underlying mechanisms. Analysis of DTI data based on a tensor model and a limited number of diffusion directions has limited accuracy in regions of crossing tracts, potentially creating false tracts (Alexander et al., 2007). Future studies could use more advanced acquisition methods, including multi-shell imaging, high-angular-resolution diffusion imaging, and neurite orientation dispersion and density imaging. In addition, to overcome the limitations of diffusion models (Wedeen et al., 2005), diffusion spectrum imaging or CSD may be superior approaches for fiber-specific modeling and network weighting. dMRI has been a popular neuroimaging technique, with a well-understood methodology to construct the structural connectome. With recent methodological advances, structural networks constructed by multimodal MRI have proved capable of predicting cognitive variation at the individual level (Seidlitz et al., 2018). It will be interesting to combine different approaches to map the changes in PD. We recommend that researchers report data in numerical form, even if the results are not statistically significant; this is crucial for secondary research. Some studies did not specify whether multiple-comparison methods in comparing statistical differences; given the potential influence on interpretation (Meskaldji et al., 2013), future studies should apply multiple-comparison corrections, and it should be explicitly stated.

Researchers will need to select more homogeneous (or stratified) samples by considering demographic variables in more detail. The accumulation of validated evidence from connectome studies will help to reveal biological markers of specific subtypes (such as PD patients with MCI). Dopamine transporter imaging (DAT-SPECT or DAT-PET) can support the clinical diagnosis of PD (Liu et al., 2018; Mirpour et al., 2018; Suwijn et al., 2015), yet only two of the 16 included studies performed DAT-SPECT and PD patients enrolled in future studies should have this scan when research costs are permitted. This meta-analysis focused on cross-sectional studies, but longitudinal studies are needed to determine how structural connectome may change in symptom remission after clinical treatment. Similar global disruptions of structural networks have been identified in other neurodegenerative disorders; for example Alzheimer's patients reportedly exhibit similarly increased L_p_ and decreased E_glob_ (Lo et al., 2010), possibly suggesting a shared profile of neurobiological changes in the neurodegenerative disorders. Further study is needed of the distinct patterns unique to specific neurodegenerative diseases.

## Conclusion

Despite the inconsistent reports of structural topological organization, our meta-analysis provides evidence of decreased network segregation (decreased C_p_) and integration (decreased E_glob_ and increased L_p_), representing a shift from a balanced small-world network to a ‘weaker small-worldization’ pattern. Abnormalities in the PD brain structural connectome provide anatomical insights into the pathogenesis of PD, and topological properties have the potential to become biomarkers of PD. This study contributes to psychoradiology (Gong et al., 2021; Li et al., 2021; Lui et al., 2016; Huang et al., 2019; Pan et al., 2021; Suo et al., 2022b), an evolving subspecialty of radiology guiding diagnostic and therapeutic decision making in neuropsychiatric disorders.

### Supplementary Information

Below is the link to the electronic supplementary material.

Supplementary file1 (DOCX 131 KB)
