# Supplementary material for: Global Alterations of Whole Brain Structural Connectome in Parkinson’s Disease: A Meta-analysis
Source: Neuropsychol Rev. 2022 Sep 20;33(4):783–802. doi: 10.1007/s11065-022-09559-y (PMC10770271; doi:10.1007/s11065-022-09559-y)
Supplement: Supplementary file 1 — Supplementary file1 (DOCX 131 KB) [file 11065_2022_9559_MOESM1_ESM.docx]

**Supplementary Material**

**Materials and Methods**

Calculations of overall mean and standard deviation of two subgroups combined. Subgroup 1 (with sample size = N_1_, mean = M_1_ and SD = SD_1_) and Subgroup 2 (with sample size = N_2_, mean = M_2_, and SD = SD_2_) (Higgins et al., 2021).

$$M_{combined}=\frac{M_{1}N_{1}{+ M}_{2}N_{2}}{N_{1}+N_{2}}$$

$${SD}_{combined}=\sqrt{\frac{\left( N_{1}-1 \right){SD}_{1}^{2}+\left( N_{2}-1 \right){SD}_{2}^{2}+\frac{N_{1}N_{2}}{N_{1}+N_{2}}(M_{1}^{2}+M_{2}^{2}-2M_{1}M_{2})}{N_{1}{+ N}_{2} - 1}}$$

**Supplementary Table S1.** Summaries of topological parameters used in this meta-analysis.

| **Network topology measure** | **Brief description** |
| --- | --- |
|
| Clustering coefficient | Ratio of the number of connections that exist between neighbors of a node to the maximum number of connections possible between these nodes, calculated for each node individually and averaged over the entire network. |
| Shortest path length | The sum of the counts (binary network) of edges or the weights (weighted network) of edges on the shortest path between any two nodes. |
| Characteristic path lengths | The average shortest path length over all pairs of nodes: the commonest functional integration metric. |
| Normalized clustering coefficient | The clustering coefficient of a network normalized to the clustering coefficient of the matching random network. |
| Normalized characteristic path length | The characteristic path length of the network normalized to the matching random network. |
| Small-worldness | The ratio of normalized clustering coefficient to normalized characteristic path length. A small-world network is defined as small-worldness > 1, normalized clustering coefficient > 1 and normalized characteristic path length ≈ 1. |
| Global efficiency | Average inverse harmonic shortest path length over all the pairs of nodes: a measure of the efficiency of parallel information transfer in the network. |
| Local efficiency | The average of the normalized sum of the inverse of the shortest path lengths in the local region, utilizing the path length information between a node and its neighbors. |
| Modularity | Quantifies the difference between the weight of intra-module links of the actual network and the random network where the connections are randomly weighted: reflects the organization of networks into modules or communities with high levels of local clustering |
| Network density (= sparsity) | An evaluation of network ‘wiring cost’: the number of existing connections divided by the total number of possible connections of all nodes (regulated by setting different thresholds). |
| Nodal strength | A centrality measure: the sum of all edge weights between the node and all other nodes in the network. |
| Network strength | The average of all the nodal strengths. |

| **Databases** | | PubMed (134), PsycINFO (103), Embase (261) and Web of Science (169) |
| --- | --- | --- |
| **Date of search** | | Initial search April 2021, updated August 2021 |
| **Search strategy (terms)** | ***Parkinson's disease*** | "parkinson's disease" OR "parkinson" |
|  | ***Graph-theoretical analysis*** | "graph theor*" OR "graph metric*" OR "graph analys*" OR "network analys*" OR "network architect*" OR "structural connect*" OR "topolog*" OR "small worldness" OR "clustering coefficient" OR "characteristic path length" OR "global path length" OR "shortest path length" OR "global efficiency" OR "local efficiency" OR "degree" OR "strength" OR "betweenness centrality" OR "modularity" OR "assortativity" |
|  | ***dMRI*** | "diffusion magnetic resonance imaging" OR "diffusion MRI" OR "dMRI" OR "diffusion weighted imag*" OR "diffusion weighted MRI" OR "diffusion tensor imaging" OR "DTI" OR "diffusion kurtosis imag*" OR "DKI" OR "diffusion spectrum imag*" OR "DSI" OR "deterministic tractography" OR "probabilistic tractography" |

**Supplementary Table S2.** Search strategy, date of searches, and number of results returned for each database used in the meta-analysis.

**Supplementary Table S3.** Quality assessment checklist.

| **Category 1: Participants Score (Full met, 1; partially met, 0.5; not met, 0)** |
| --- |
| 1. Patients were evaluated prospectively, specific diagnostic criteria were applied, and demographic data was reported.  2. Healthy controls were evaluated prospectively, psychiatric and medical illnesses were excluded, and demographic data was reported.  3. Important variables (e.g. illness duration, medication status, UPDRS Ⅲ score, and H&Y stage) were checked either by stratification or statistically.  4. Sample size per group (including subgroup if necessary) > 10, and there were no significant between-group differences in age and sex. |
| **Category 2: Methods for image acquisition and analysis** |
| 5. Magnet strength at least 1.5T.  6. dMRI with at least 12 directions was used.  7. The imaging technique was clearly enough described to be reproduced.  8. The parcellation procedure was clearly enough described to be reproduced, and no brain regions were excluded (e.g., cerebellum and ventricles).  9. The definition of edge and calculation of weighted or binary network were clearly enough described to be reproduced.  10. Calculation of graph metrics were clearly enough described to be reproduced. |
| **Category 3: Results and conclusions** |
| 11. Corrections for multiple comparisons (if necessary).  12. Statistical parameters provided for significant and important non-significant differences.  13. Conclusions were consistent with the results obtained and the limitations were discussed. |
| **TOTAL** /13 |

Abbreviations: dMRI, diffusion magnetic resonance imaging; H&Y, Hoehn and Yahr stage; UPDRS, Unified Parkinson's Disease Rating Scale.

**Supplementary Table S4.** Results of the subgroup analyses.

| Outcome | Subgroup | Variable | K | Hedges’ g | Lower limit | Upper limit | Z-Value | P-Value | I^2^ (%) |
| --- | --- | --- | --- | --- | --- | --- | --- | --- | --- |
| C_p_ | Medication status | Drug-naive/Off-state | 6 | -0.562 | -1.078 | -0.046 | -2.136 | 0.033* | 86.878 |
|  |  | On-state | 6 | -0.261 | -0.476 | -0.046 | -2.383 | 0.017* | 36.010 |
|  | Tractography | DT | 10 | -0.277 | -0.556 | 0.002 | -1.946 | 0.052 | 77.372 |
|  |  | PT | 3 | -0.652 | -1.251 | -0.052 | -2.130 | 0.033* | 75.716 |
|  | Weighted methods | FA | 4 | -0.232 | -0.453 | -0.011 | -2.059 | 0.040* | 27.444 |
|  |  | NOS | 4 | -0.437 | -1.021 | 0.147 | -1.465 | 0.143 | 80.804 |
|  | No. of directions | ≥ 30 | 8 | -0.323 | -0.561 | -0.085 | -2.658 | 0.008* | 61.068 |
|  |  | < 30 | 3 | -0.626 | -1.865 | 0.612 | -0.991 | 0.322 | 94.027 |
|  | Threshold | Absolute | 7 | -0.332 | -0.757 | 0.093 | -1.529 | 0.126 | 84.178 |
|  |  | Sparsity | 4 | -0.508 | -0.948 | -0.069 | -2.267 | 0.023* | 74.750 |
|  | Atlas | AAL | 7 | -0.281 | -0.686 | 0.123 | -1.362 | 0.173 | 84.079 |
|  |  | Non-AAL | 6 | -0.446 | -0.712 | -0.180 | -3.286 | 0.001 * | 52.429 |
| L_p_ | Medication status | Drug-naive/Off-state | 5 | 0.382 | 0.195 | 0.568 | 4.006 | < 0.001* | < 0.001 |
|  |  | On-state | 7 | 0.132 | -0.191 | 0.455 | 0.800 | 0.424 | 71.701 |
|  | Tractography | DT | 10 | 0.289 | 0.120 | 0.459 | 3.354 | 0.001* | 39.440 |
|  |  | PT | 3 | 0.023 | -0.689 | 0.736 | 0.064 | 0.949 | 77.666 |
|  | Weighted methods | FA | 4 | 0.365 | 0.179 | 0.550 | 3.855 | < 0.001* | < 0.001 |
|  |  | NOS | 4 | 0.175 | -0.320 | 0.669 | 0.693 | 0.489 | 68.061 |
|  | No. of directions | ≥ 30 | 8 | 0.332 | 0.139 | 0.526 | 3.374 | 0.001* | 37.388 |
|  |  | < 30 | 3 | 0.357 | 0.078 | 0.637 | 2.505 | 0.012* | < 0.001 |
|  | Threshold | Absolute | 8 | 0.303 | 0.107 | 0.499 | 3.025 | 0.002* | 29.942 |
|  |  | Sparsity | 3 | 0.420 | 0.197 | 0.643 | 3.693 | < 0.001* | < 0.001 |
|  | Atlas | AAL | 8 | 0.290 | 0.102 | 0.478 | 3.020 | 0.003 * | 30.180 |
|  |  | Non-AAL | 5 | 0.220 | -0.149 | 0.588 | 1.167 | 0.243 | 72.198 |
| σ | Medication status | Off-state | 2 | 0.274 | -0.077 | 0.626 | 1.528 | 0.126 | < 0.001 |
|  |  | On-state | 5 | -0.162 | -0.552 | 0.229 | -0.812 | 0.417 | 77.015 |
|  | Tractography | DT | 6 | 0.052 | -0.144 | 0.247 | 0.515 | 0.606 | 22.239 |
|  |  | PT | 2 | -0.326 | -1.735 | 1.082 | -0.454 | 0.650 | 93.176 |
|  | Weighted methods | FA | 2 | 0.153 | -0.139 | 0.444 | 1.026 | 0.305 | < 0.001 |
|  |  | NOS | 4 | 0.0003 | -0.536 | 0.537 | 0.001 | 0.999 | 81.104 |
|  | No. of directions | ≥ 30 | 5 | -0.036 | -0.425 | 0.352 | -0.184 | 0.854 | 75.632 |
|  |  | < 30 | 2 | 0.245 | -0.078 | 0.567 | 1.488 | 0.137 | < 0.001 |
|  | Threshold | Absolute | 6 | 0.171 | 0.005 | 0.338 | 2.016 | 0.044* | < 0.001 |
|  |  | Sparsity | 1 | -1.069 | -1.705 | -0.433 | -3.296 | 0.001* | < 0.001 |
|  | Atlas | AAL | 4 | 0.130 | -0.088 | 0.349 | 1.170 | 0.242 | 0.000 |
|  |  | Non-AAL | 4 | -0.192 | -0.712 | 0.329 | -0.722 | 0.470 | 82.671 |
| E_loc_ | Medication status | Off-state | 5 | -0.124 | -0.352 | 0.103 | -1.071 | 0.284 | < 0.001 |
|  |  | On-state | 2 | -0.238 | -0.520 | 0.043 | -1.658 | 0.097 | < 0.001 |
|  | Tractography | DT | 6 | -0.192 | -0.366 | -0.018 | -2.165 | 0.030* | < 0.001 |
|  |  | PT | 2 | 0.154 | -0.318 | 0.626 | 0.640 | 0.522 | < 0.001 |
|  | Weighted methods | FA | 4 | -0.102 | -0.378 | 0.174 | -0.724 | 0.469 | 10.675 |
|  |  | NOS | 2 | -0.094 | -0.413 | 0.224 | -0.580 | 0.562 | < 0.001 |
|  | No. of directions | ≥ 30 | 6 | -0.193 | -0.383 | -0.004 | -2.004 | 0.045* | < 0.001 |
|  |  | < 30 | 2 | -0.027 | -0.349 | 0.294 | -0.166 | 0.868 | < 0.001 |
|  | Threshold | Absolute | 7 | -0.105 | 0.092 | 0.008 | -0.285 | 0.075 | < 0.001 |
|  |  | Sparsity | 1 | -0.363 | 0.197 | 0.039 | -0.750 | 0.024* | < 0.001 |
|  | Atlas | AAL | 5 | -0.168 | -0.358 | 0.022 | -1.730 | 0.084 | 0.000 |
|  |  | Non-AAL | 3 | -0.084 | -0.441 | 0.274 | -0.459 | 0.646 | 16.992 |
| E_glob_ | Medication status | Drug-naive/Off-state | 8 | -0.332 | -0.601 | -0.063 | -2.422 | 0.015* | 58.094 |
|  |  | On-state | 4 | -0.469 | -0.679 | -0.259 | -4.385 | < 0.001* | < 0.001 |
|  | Tractography | DT | 9 | -0.370 | -0.539 | -0.202 | -4.299 | < 0.001* | 33.008 |
|  |  | PT | 4 | -0.271 | -0.805 | 0.262 | -0.998 | 0.318 | 66.593 |
|  | Weighted methods | FA | 6 | -0.236 | -0.500 | 0.027 | -1.759 | 0.079 | 51.576 |
|  |  | NOS | 4 | -0.399 | -0.712 | -0.086 | -2.499 | 0.012* | 34.971 |
|  | No. of directions | ≥ 30 | 10 | -0.325 | -0.502 | -0.149 | -3.609 | < 0.001* | 34.640 |
|  |  | < 30 | 3 | -0.522 | -1.050 | 0.005 | -1.940 | 0.052 | 69.957 |
|  | Threshold | Absolute | 9 | -0.350 | -0.568 | -0.131 | -3.141 | 0.002* | 46.050 |
|  |  | Sparsity | 4 | -0.380 | -0.692 | -0.069 | -2.395 | 0.017* | 51.072 |
|  | Atlas | AAL | 7 | -0.327 | -0.535 | -0.119 | -3.079 | 0.002 * | 40.996 |
|  |  | Non-AAL | 6 | -0.391 | -0.693 | -0.089 | -2.540 | 0.011 * | 49.692 |

Abbreviations: AAL, automated anatomic labeling; C_p_, clustering coefficient; DT, deterministic tractography; E_glob_, global efficiency; E_loc_, local efficiency; FA, fractional anisotropy; L_p_, characteristic path length; NOS, number of streamlines; PT, probabilistic tractography; σ, Small-worldness; *, significant at p < 0.05.

**Supplementary Table S5.** Results of the meta-regression analysis.

| Outcome | Moderator | No. of studies | Regression coefficient | Standard error | Lower limit | Upper limit | T-Value | P-Value | R^2^ |
| --- | --- | --- | --- | --- | --- | --- | --- | --- | --- |
| C_p_ | UPDRS-III | 13 | 0.046 | 0.023 | -0.003 | 0.096 | 2.060 | 0.064 | 0.158 |
|  | H&Y stages | 10 | 0.485 | 0.367 | -0.360 | 1.331 | 1.320 | 0.222 | 0.012 |
|  | Duration | 12 | 0.101 | 0.076 | -0.069 | 0.270 | 1.320 | 0.215 | 0.003 |
|  | Age | 13 | 0.040 | 0.060 | -0.091 | 0.172 | 0.670 | 0.515 | < 0.001 |
|  | Sex (male %) | 12 | -5.217 | 1.765 | -9.150 | -1.284 | -2.960 | 0.014* | 0.443 |
| L_p_ | UPDRS-III | 12 | -0.002 | 0.013 | -0.032 | 0.028 | -0.140 | 0.891 | < 0.001 |
|  | Duration | 11 | -0.058 | 0.047 | -0.165 | 0.049 | -1.230 | 0.249 | 0.114 |
|  | Age | 12 | -0.056 | 0.025 | -0.113 | 0.0002 | -2.220 | 0.051 | 0.714 |
|  | Sex (male %) | 11 | 1.557 | 1.065 | -0.851 | 3.965 | 1.460 | 0.178 | < 0.001 |
| E_glob_ | UPDRS-III | 13 | 0.015 | 0.015 | -0.018 | 0.048 | 1.020 | 0.331 | < 0.001 |
|  | H&Y stages | 10 | 0.034 | 0.185 | -0.391 | 0.460 | 0.190 | 0.857 | < 0.001 |
|  | Duration | 12 | 0.049 | 0.050 | -0.061 | 0.160 | 0.990 | 0.344 | < 0.001 |
|  | Age | 13 | 0.011 | 0.046 | -0.089 | 0.112 | 0.250 | 0.807 | < 0.001 |
|  | Sex (male %) | 13 | -1.032 | 1.260 | -3.805 | 1.741 | -0.820 | 0.430 | < 0.001 |

Abbreviations: H&Y, Hoehn and Yahr stage; UPDRS, Unified Parkinson's Disease Rating Scale; C_p_, clustering coefficient; E_glob_, global efficiency; L_p_, characteristic path length; *, significant at p < 0.05.

**Supplementary Fig S1.** Publication bias of graph theoretical measures

**A.** Publication bias of clustering coefficient. **B.** Publication bias of local efficiency.

**C.** Publication bias of characteristic path length. **D.** Publication bias of global efficiency.

**E.** Publication bias of small-worldness.

**Supplementary Fig S2.** Sensitivity analysis of graph theoretical parameters using the leave-one-out method.

1. Sensitivity analysis of clustering coefficient

**B.** Sensitivity analysis of normalized clustering coefficient

**C.** Sensitivity analysis of local efficiency

**D.** Sensitivity analysis of characteristic path length

**E.** Sensitivity analysis of normalized characteristic path length

**F.** Sensitivity analysis of global efficiency

**G.** Sensitivity analysis of small-worldness

**H.** Sensitivity analysis of density

**I.** Sensitivity analysis of strength

**J.** Sensitivity analysis of modularity

**References**

Higgins, J., Thomas, J., Chandler, J., Cumpston, M., Li, T., Page, M., & Welch, V. ( 2021). Cochrane Handbook for Systematic Reviews of Interventions version 6.2 (updated February 2021). Cochrane, 2021. from Available from: <www.training.cochrane.org/handbook>
